# Home-based Computer Assisted Arm Rehabilitation (hCAAR) robotic device for upper limb exercise after stroke: results of a feasibility study in home setting

**DOI:** 10.1186/1743-0003-11-163

**Published:** 2014-12-12

**Authors:** Manoj Sivan, Justin Gallagher, Sophie Makower, David Keeling, Bipin Bhakta, Rory J O’Connor, Martin Levesley

**Affiliations:** Academic Department of Rehabilitation Medicine, University of Leeds, Leeds, LS1 3EX UK; School of Mechanical Engineering, University of Leeds, Leeds, LS1 3EX UK; National Demonstration Centre in Rehabilitation Medicine, Leeds, UK

**Keywords:** Cerebrovascular, Robot, Rehabilitation, Tele-rehabilitation

## Abstract

**Background:**

Home-based robotic technologies may offer the possibility of self-directed upper limb exercise after stroke as a means of increasing the intensity of rehabilitation treatment. The current literature has a paucity of robotic devices that have been tested in a home environment. The aim of this research project was to evaluate a robotic device Home-based Computer Assisted Arm Rehabilitation (hCAAR) that can be used independently at home by stroke survivors with upper limb weakness.

**Methods:**

hCAAR device comprises of a joystick handle moved by the weak upper limb to perform tasks on the computer screen. The device provides assistance to the movements depending on users ability. Nineteen participants (stroke survivors with upper limb weakness) were recruited. Outcome measures performed at baseline (A0), at end of 8-weeks of hCAAR use (A1) and 1 month after end of hCAAR use (A2) were: Optotrak kinematic variables, Fugl Meyer Upper Extremity motor subscale (FM-UE), Action Research Arm Test (ARAT), Medical Research Council (MRC) and Modified Ashworth Scale (MAS), Chedoke Arm and Hand Activity Inventory (CAHAI) and ABILHAND.

**Results:**

Two participants were unable to use hCAAR: one due to severe paresis and the other due to personal problems. The remaining 17 participants were able to use the device independently in their home setting. No serious adverse events were reported. The median usage time was 433 minutes (IQR 250 – 791 min). A statistically significant improvement was observed in the kinematic and clinical outcomes at A1. The median gain in the scores at A1 were by: movement time 19%, path length 15% and jerk 19%, FM-UE 1 point, total MAS 1.5 point, total MRC 2 points, ARAT 3 points, CAHAI 5.5 points and ABILHAND 3 points. Three participants showed clinically significant improvement in all the clinical outcomes.

**Conclusions:**

The hCAAR feasibility study is the first clinical study of its kind reported in the current literature; in this study, 17 participants used the robotic device independently for eight weeks in their own homes with minimal supervision from healthcare professionals. Statistically significant improvements were observed in the kinematic and clinical outcomes in the study.

**Electronic supplementary material:**

The online version of this article (doi:10.1186/1743-0003-11-163) contains supplementary material, which is available to authorized users.

## Introduction

Stroke is a major public health problem with an annual incidence estimate of 15 million people worldwide [1], between 200 and 300 per 100,000 people in Europe [2] and around 130,000 in the United Kingdom (UK) [3]. Globally, it is the third leading cause of mortality (after coronary heart disease and cancer) and results in 5 million deaths annually [4]. Stroke is the leading cause of adult onset disability worldwide, and annually, leads to 5 million people developing long-term disability and dependency [1, 2, 4]. In the UK, the estimated direct and indirect costs of stroke care are £ 9 billion a year, accounting for approximately 5% of the total National Health Service (NHS) costs [3]. With a progressively ageing population and improved stroke survival rates, the number of survivors with disability is expected to increase in the coming decades.

Up to 85% of survivors experience some degree of paresis of the upper limb at the onset [5] and only 20% to 56% of survivors regain complete functional use of the affected upper limb in spite of therapeutic intervention at 3 months [6–9]. Recovery of upper limb function is generally slower and less complete than return of mobility. This is partly due to the complexity of movement required for upper limb function [10, 11]. Motor recovery has been shown to be the most influential factor in determining well-being one year after stroke [12] and hence the emphasis of rehabilitation interventions is to improve upper limb function and reduce long term disability [9].

Novel robotic technology can provide repetitive meaningful tasks, greater intensity of practice, stimulating and engaging environment for user and alleviate the labour-intensive aspects of hands-on conventional therapy. There are a number of complex robotic devices like Massachusetts Institute of Technology (MIT-Manus), Mirror Image Movement Enabler (MIME), Bi-Manu-Track, Assisted Rehabilitation and Measurement (ARM) Guide, ARMin, GENTLE system, intelligent Pneumatic Arm Movement (iPAM) and others that have been developed over the last two decades to assist upper arm training in rehabilitation [13, 14]. Meta-analysis studies have observed that robotic therapy can be as effective as conventional therapy in improving motor strength and functional ability [15, 16].

Micera et al. have put forward a simple hierarchical system of classifying robotic devices for upper limb rehabilitation after stroke 1) Exoskeleton devices with greater range of movement and complex design suited for use in hospitals and research labs for users with severe disability 2) Operational devices which are less complex, end-effector and suitable for use by users with moderate disability [17]. The operational devices group can be further sub-classified as a) Class 1 devices that have low mechanical friction, high back-driveability, fine tuned visco-elastic properties and high cost that can be used in lab setting and b) Class 2 devices that have a simple mechanical structure, compensation of inertia/friction, no back-drivability and low cost to be used in telerehabilitation setting at home. In the current literature, there is a plethora of exoskeleton and class 1 devices manufactured and tested so far. However there is an obvious paucity of class 2 devices that have been tested in home setting. There is a clear need to explore the challenges of making low-cost home-based robots that are simple, acceptable and effective in improving arm function. The number of people needing arm rehabilitation post-stroke is increasing worldwide and there is growing emphasis of moving rehabilitation resources to community-setting and peoples homes.

The technical challenges of home-based robotic therapy are to make the technology safe to be deployed and usable in a home setting. The footprint of the device needs to be acceptable to the patient, family and carers. The user should be able to easily set-up and use the device without the therapist being present for each session. The user would need access to engineering support for technical issues and would need to be remotely supervised by a therapist to ensure appropriate therapy is being delivered. The clinical challenges are many; the technology needs to be able to match conventional therapy principles and provide the relevant therapy to the user. There is a risk of dehumanisation of the rehabilitation therapy if there is little interaction with the therapist and other patients. The therapy will need to address personal functional needs and will need to be tailor-made for each individual user.

There have been a few devices developed to provide home-based robotic upper limb rehabilitation for stroke patients. RUPERT is a wearable exoskeleton robot that helps direct the upper limb to perform functional activities in a three-dimensional virtual reality [18]. It has been tested in the home setting in two chronic stroke subjects with improvement in the accuracy and smoothness of their movements [18]. The impact on daily activities was not reported. The exoskeleton needs to be fitted to the user’s upper limb by the family member or carer. The acceptability of the device needs to be tested in a larger heterogeneous sample of stroke subjects in a home setting.

Johnson et al. [19] developed an upper limb stroke therapy suite (intended for home use) consisting of affordable hardware platforms, such as the force-reflecting joystick (Therajoy) and wheel (TheraDrive) working on a customisable universal software platform (UniTherapy). A sample of 16 chronic stroke subjects with mild to moderate upper limb weakness tried the system; simultaneous EMG recording of the upper limb muscles demonstrated that the robot therapy can be personalised in terms of the muscles targeted or activated by using a choice of joystick and wheel tasks. The system also has the ability to accurately track movement kinematics that can be useful to monitor progress. The system is yet to be tested in a clinical study in a home setting.

The Java therapy system is based on wrist exercises using a low-cost commercial force feedback joystick connected to a customised computer program of therapeutic activities available on the web [20]. The system has been designed for home use and the therapy can be monitored remotely by a therapist using a low-cost web camera and teleconferencing software. One stroke survivor used the system for a 12-week period and showed improvements in movement speed and movement control. The low-cost system (estimated to cost $240 for the joystick, upper limb rest, splint and base) received high satisfaction scores from the user and his carer. This is yet to be tested in a larger clinical study in the home setting.

Wood et al. have developed a simple ‘Palanca’ sliding lever device used to play an electronic ping-pong game on the computer and have shown improvement in the functional abilities of four stroke subjects after using the device [21]. This feasibility study was conducted in the research centre and showed that the low-cost device helped maintain high level of interest, motivation and enjoyment in therapy. A larger scale study in the home setting is being planned.

The Assisted Movement with Enhanced Sensation (AMES) device provides assistance to uniaxial flexion and extension movements of the wrist (and fingers) or ankle joint. The device also provides vibration sensation to the antagonist muscle-stretching tendon when the agonist muscle is performing the desired action to provide somatosensory feedback during movement. The device provides visual feedback on the torque exerted by the user. A study of upper limb exercises in the home setting, involving eight chronic stroke participants, showed improvements in the strength and range of movement in the wrist and fingers after six months of home use. The effect on functional abilities of the upper limb was not reported. During the home-use period, three participants needed additional training with EMG feedback in the research laboratory as they could not generate adequate torque to be able to use the device. The system lacks variation in tasks that can affect long-term usage (engagement in therapy) and this needs to be explored in a larger sample of patients [22].

The purpose of this study was to test the feasibility of stroke survivors using a low-cost restorative rehabilitation robotic system, home-based Computer Assisted Arm Rehabilitation (hCAAR), to undertake independent upper limb exercises at home. The aims were to test whether a) hCAAR could be safely used in a home setting with minimal supervision and b) whether using the device improved upper limb movement and function.

## Methods

### hCAAR device

**Figure 1:**
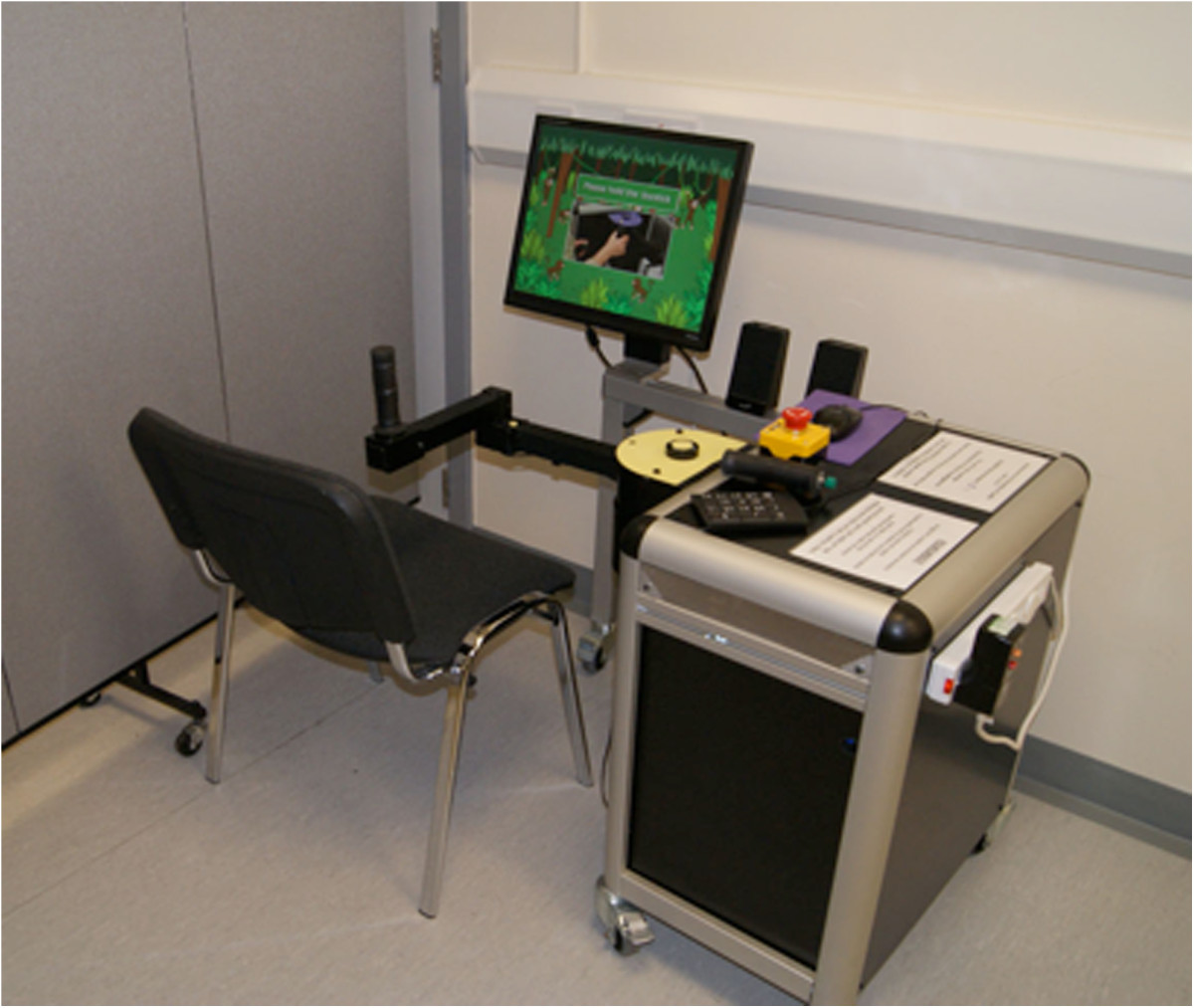
**hCAAR device.**

The hardware components of hCAAR consist of a Personal Computer (PC) allowing interaction between the user and the computer software (Figure 1). The interface equipment consists of a joystick handle linked to a chassis. The chassis allows the handle to move within a set workspace relative to the user. The exercise workspace can be adjusted physically by changing the lengths of the robot arms, or in software by scaling the difference between the movements of the joystick to pixels on the screen. In this study the workspace was maintained as a constant for all users. The motion of the device is limited to a two-dimensional plane at the central attachment point of the joystick.

The whole device is designed to be portable enough to be deployed in a home setting and not to require any additional equipment or furniture other than a chair for the user to sit on. hCAAR consists of a base unit housing the chassis and PC (39.9 kg) with a screen and stand (5.2 kg), the robot arm (6.8 kg) and a supporting leg (2.6 kg). The footprint of the assembled device is 65 cm by 95 cm and it is 120 cm high.

The interface device has a system of motors and pulleys that provide assistance to the motion of the joystick handle. The mechanism is driven by motors rated to 170mNm of continuous torque, which delivers a continuous torque after gearing of 14.2 Nm at the joystick’s shoulder and 16.41 Nm at the joystick’s elbow. This can produce a maximal excursion of forward flexion and extension of 70° at the shoulder and 320° of flexion and extension at the elbow of the joystick. Encoders on the motors allow tracking of the joystick handle position. This creates a position control loop, which can be changed in real time to make the handle move to different positions in the exercise workspace. Within the chassis a controller, motor and gear system enable force feedback from the software to guide the position of the handle. All of the above components are covered by purpose-built panels and set-up in a moveable trolley system.

**Figure 2:**
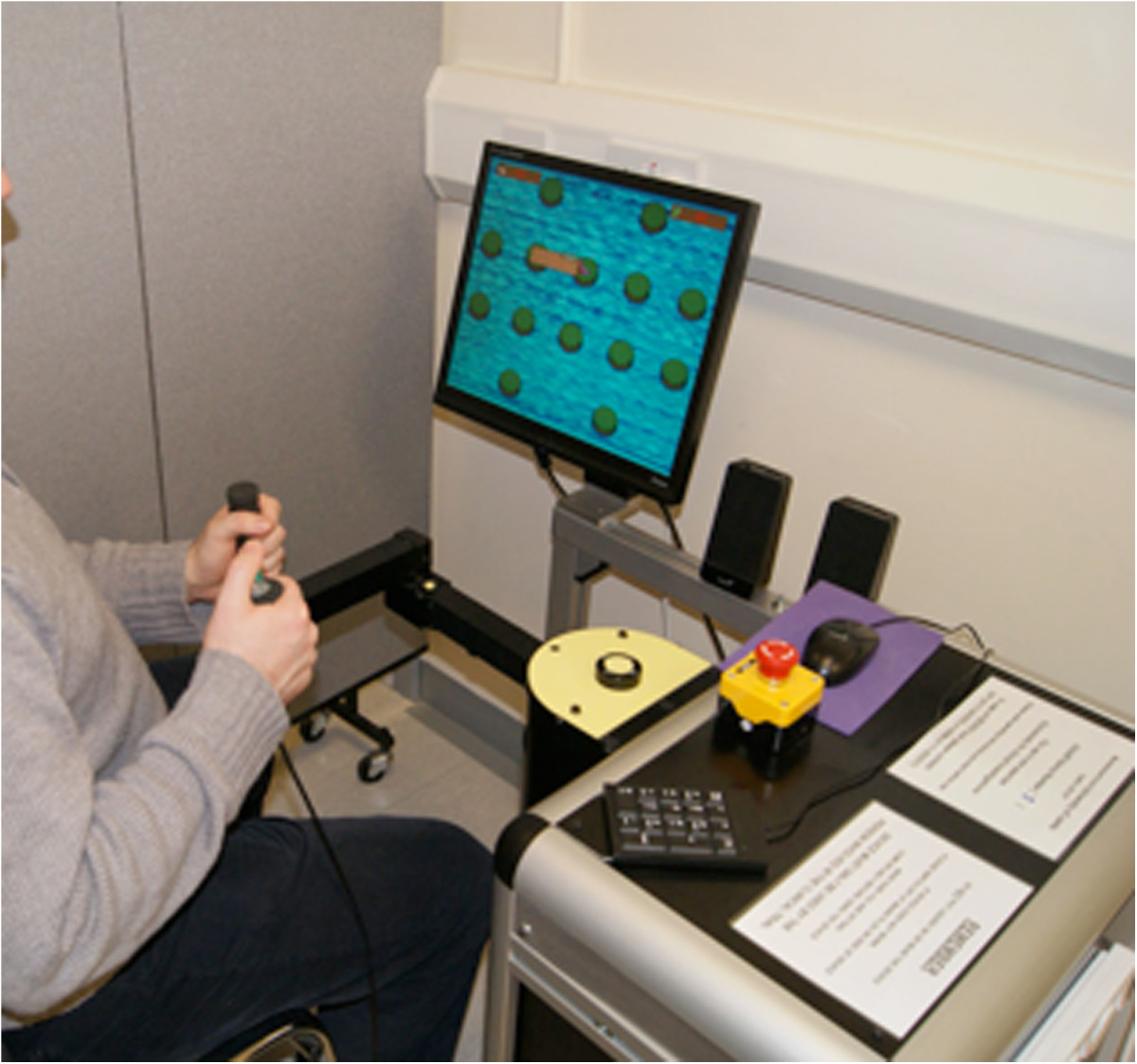
**A left-hand device being used.**

There is an additional button switch connected to the computer that is operated by the unaffected arm while choosing menu items and interacting with objects when playing the computer game (Figure 2). An emergency stop button in the system enables the user to disconnect the motor assistance to the joystick in case of emergency. The device is purpose-built for use on one side only so that the joystick handle is operated by affected/impaired arm and the unaffected arm is able to operate the switch and keypad. The unaffected arm can be used to operate the emergency button if needed. The equipment was tested for reliability and physical and electrical safety.

The computer program includes a base “software platform” for underlying functions and a set of activity-based tasks that was used to direct and control the exercises. The software also measures the number of hits in the assessment exercise. The assistance levels will adjust according to the performance in the assessment exercise. As the user performance increases, the assistance levels decrease by the set algorithm of the software. The computer screen provides visual and auditory feedback of the target location and the movement of the joystick. The baseline clinical examination and computer assessment exercise allows the initial exercise parameters (duration, nature of games, game levels and assistance level) to be set.

There are two operational modes for the device:

- Active nonassist – the movement is performed completely by individual’s own effort with no assistance/resistance offered by the device. This mode is used for the assessment exercise prior to game play.
- Active assisted bimanual mode – the individual initiates the movement and is aided by the device towards the goal. The joystick directed movement on the monitor can complete the task only when accompanied by the action of a switch device controlled by the unaffected arm. This mode is used during game play.

There are eight computer games that are designed to provide arm exercises to the participant. Each game involves a series of linear movements within the monitor workspace to be performed by moving the joystick using the affected arm. The characters have to be moved to the target and the switch device pressed by the unaffected arm to complete each component task within the game. Four games have animated characters to provide more fun while performing tasks and four games do not have the animated characters. Each game is based on a series of movement steps on the screen. Each game has 75 built-in levels designed to provide a hierarchical order of difficulty in terms of the number of movement steps and the extent of workspace used. For example level 1 of the chase game has a small workspace of approximately 4 × 2 inches on the screen whereas level 75 has a workspace of approximately 6 × 6 inches on the screen. The range of movement the shoulder and elbow go through while performing level 75 is greater than the range used in level 1. This makes the levels progressively difficult and more challenging to the user.

### Study design

**Figure 3:**
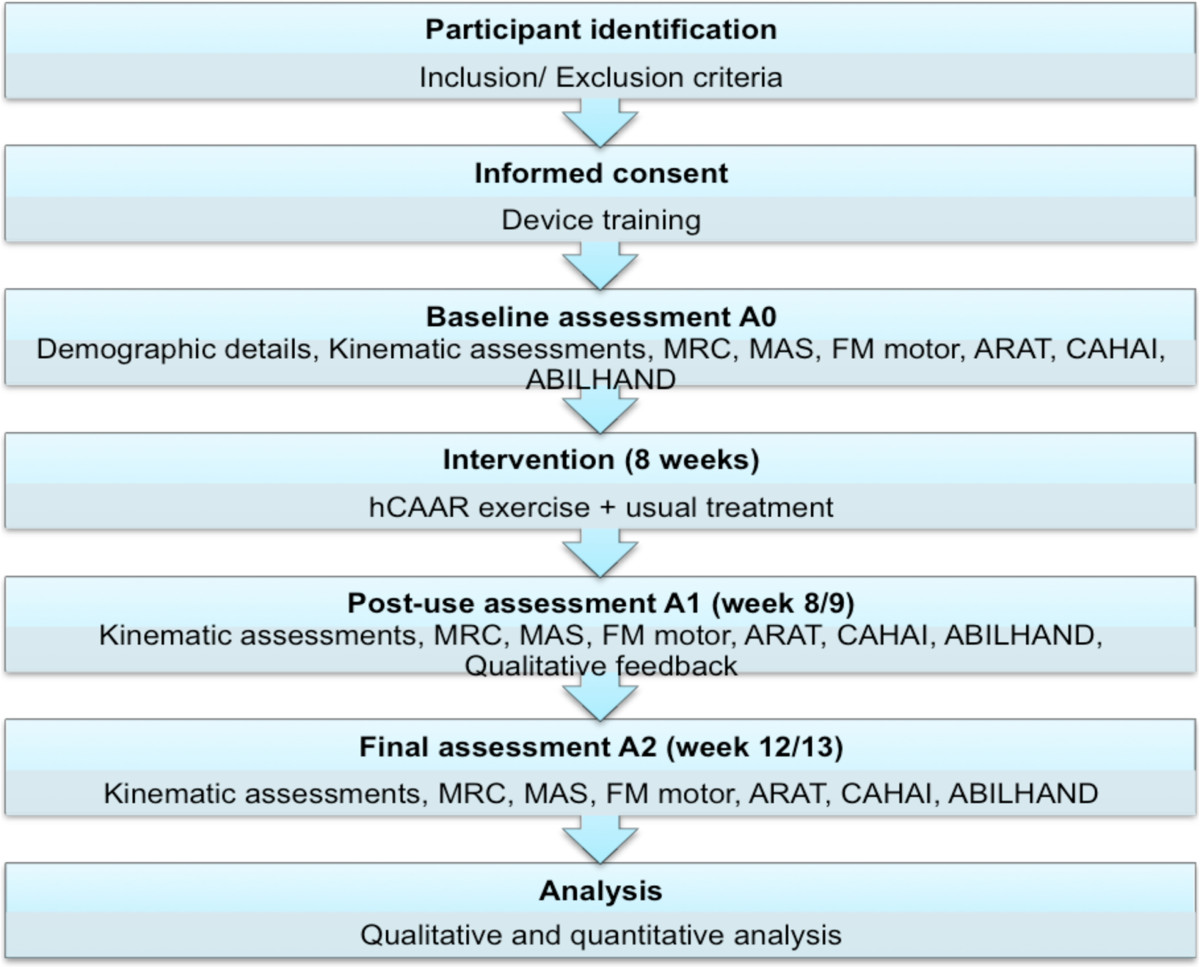
**Feasibility study flow diagram.**

This was a pilot open label cohort phase 2 clinical study as defined by the MRC Guidelines on Complex Interventions [23] for a new restorative rehabilitation device. The study involved 8 weeks of home arm exercise using hCAAR for stroke survivors with residual upper limb weakness. This was not a randomised control study, all consented participants received the hCAAR system to undertake home exercises in addition to their usual treatments. The usual treatment varied between individual participants and involved treatments such as NHS community physiotherapy, private physiotherapy or self-exercise. The study plan is as shown in Figure 3.

The study had approvals from the National Research Ethics Committee (NREC), local Research and Development (R&D) department and the Medicines and Healthcare products Regulatory Agency (MHRA). People with stroke admitted to stroke rehabilitation inpatient services or stroke survivors attending outpatient clinics within a large University Hospital and a primary care trust were screened for suitability for the study.

The inclusion criteria were a) Age more than 18 years b) Diagnosis of ischaemic or hemorrhagic stroke at least 1 month prior to inclusion c) Residual weakness of upper limb and d) A mimimum of some voluntary arm movement to perform the hCAAR exercise tasks including suitable hand grip function. Participant in a sitting position must be able to actively move the affected hand, rested on table, by at least 15 cm. The exclusion criteria were a) Significant pain in the weak upper limb b) Significant limitation in the range of motion of weak upper limb c) Cognitive impairment affecting capacity to consent d) Sensory impairment affecting ability to use hCAAR system and e) Significant medical co-morbidities like uncontrolled epilepsy. Written consent was obtained from each participant enrolled in the study. If the participant was able to provide fully informed consent but was unable to sign or otherwise mark the consent form, provision for completion of the consent form by a family member was made.

hCAAR device was set up in the participant’s home by the research team. The general recommendation was for at least half an hour of exercise every day for at least five days every week, but it was suggested to participants that they could use the device as much as they wanted in the 8-week period. The participant’s usual medical and rehabilitation treatment continued as part of routine care and was not altered due to participation in the study. A member of the research team contacted the participant by telephone once every two weeks to check participant’s progress and to discuss any queries the participant had. At end of week 8, members of the research team visited the participant’s home to retrieve the system.

### Outcome measures

We used validated outcome measures to capture quality of arm movements and clinical/ functional effect. hCAAR usage is reported in terms of total usage time of the device during the 8-week period, this includes the assessment exercises, warm-up exercises and game play. Detailed user feedback about the device and their recommendations for future development of device were gathered by semi-structured interviews, which are not discussed in this paper.

Measurement of voluntary upper limb movement using kinematic variables was undertaken while performing a standardised reaching task similar to that reported in current literature [24, 25]. A suite of Optotrak and Optokat systems installed in a research laboratory was used to record kinematic variables of arm movement like movement time, path length and jerk. The Optotrack (Certus) system comprised of a position sensor/camera mounted on roof that captures infrared signals from the markers/diodes attached to the body of the participant being tested. These signals are sent to a system control unit that calculates the position of marker in 3 coordinates (x,y and z) and sends the 3D raw data of marker position to the host computer for further analysis. The Optokat system is a standardised seating system for the participant, comprising of a chair, frame, starting point and target touch screen. The four corners of the frame, seat, starting position and end point are attached with infrared light-emitting diodes or markers. The participant was sat in a standard position and held a stylus in the affected arm. Markers (either single or in a rigid body group) were attached to the participant’s trunk and arm at standard positions: sternum, shoulder, arm, elbow forearm and wrist.

The trial began with the stylus positioned at the start point with the elbow angle approximately 90 degrees and forearm in midprone/neutral position and wrist in neutral position. An auditory signal (which corresponds to initialisation of data collection) indicated that the participant should start the movement. Each participant was instructed to aim towards a light switch picture on a touch screen quickly and accurately, with the ultimate goal of the task to press the switch on the screen with the stylus. On touching the target, there was a bulb image displayed with auditory signal indicating end of task. The first task was reaching a near-reach target 120 mm away from the start position. The second task was a far-reach task with the target 150 mm away from start position. Five repeated trails for each task were performed and software generated kinematic data in terms of movement time, path length and normalised jerk. Movement Time (MT) is the time taken (in seconds) to complete the task of reaching from start position to the target on the screen. Path Length (PL) is the distance (in millimetres) taken to reach the target on the screen from the start position. Jerk is the rate of change of acceleration during movement and is a measure of the smoothness and efficiency of the movement. As jerk varies with movement time and distance travelled during the movement, normalising the quantity in time and distance gives the Normalised Jerk (NJ) value. NJ is a dimensionless number that allows movements of different durations and lengths to be compared [26].

The Fugl Meyer - Upper Extremity subsection (FM-UE), a measure of upper limb impairment, was used as a measure of movement ability of the affected upper limb. It is a validated measure with 33 items and a score ranging from 0 – 66 points [27]. The measure is widely used in robotic rehabilitation research and has good reliability and validity in stroke population [28–30]. One of the criticisms of the scale has been its moderate responsiveness in sub-acute and chronic stages after stroke [31, 32]. The minimal clinically important difference (MCID) is estimated to be 6.6 [33, 34].

The Action Research Arm Test (ARAT), an outcome measure of upper limb function, was used as a measure to complement the FM-UE scale and measure grasp, grip, pinch and gross movements. The 19-item scale has a score ranging from 0 to 57. Studies have reported high reliability and validity in stroke patients [33, 35]. The scale is more responsive than FM-UE [33] but is limited by its high floor and ceiling effects [36]. The MCID is estimated to be 6 [33, 37].

The Modified Ashworth Scale (MAS) was used to measure spasticity (muscle stiffness) in the paretic upper limb. Spasticity in the shoulder abductors, adductors, flexors and extensors; elbow flexors and extensors; wrist flexors and extensors; finger flexors and extensors was recorded. The MAS score (0–4) of all muscles were summated to get a total MAS score that ranged from 0 to 40 [38, 39]. The MCID for total MAS is unknown.

The Medical Research Council (MRC) scale was used to record muscle power in different muscle groups of paretic arm. Each of the above muscle groups was scored on a six-point ordinal scale 0–5 and the total MRC score ranged from 0 to 50 [40]. This method of adding scores to give a total motor power scale has been described in the literature [41].

The Chedoke Arm and Hand Activity Inventory (CAHAI, version 13.0) was used to capture the functional ability in daily activities and contribution of affected upper limb in 13 real life bilateral activities. Its score ranges between 13 and 91 [42]. It has high reliability and shown to be more responsive than ARAT in stroke patients [43]. The MCID is estimated to be 6.3 [43].

The self-reported questionnaire ABILHAND was used to capture participant perception of performance in actual daily life activities [44]. The score ranges from 0 – 46. The MCID of the scale is yet to be researched but based on the 10% rule can be estimated to be around 5 [45, 46]. The scale has been validated based on the Rasch model and gives a linear measure of manual ability as well. The responses were entered in to an online computer program (http://www.rehab-scales.org/abilhand-rasch-analysis-chronic-stroke.html) that gave the score in logits.

### Assessment schedule and blinding

A baseline assessment A0 was carried out just before home installation of the device (week 0). A post-use assessment (A1) was carried out just after completion of the 8-week usage period (week 8/9) and a final assessment A2 assessment was carried out 4-weeks after A1 (week 12/13). MS and JG/DK did the kinematic measurements in all assessments. MS assessed the clinical scores in assessment A0. SM assessed the clinical scores in A1 and A2. MS conducted and recorded the qualitative feedback interviews during A1 assessment. SM was not aware of the baseline assessment scores while assessing participants in A1 and A2. This blinding was done to minimise the assessor bias of knowing scores before intervention and being influenced by participant’s impressions of the intervention.

### Data analysis

The output of the Optotrak software was saved as an Excel file that was extracted to a master Excel file. The software provided data on movement time, peak speed, time to peak speed, path length, path length ratio, peak elbow angle and peak trunk angle. The best three trials for each task (near reach or far reach) were selected based on the shortest movement time (and selected by path length if the movement time was the same for two trials). The selection of the three best trials enabled the minimising of the bias of variation in the individual initiating the movement on the start command and dealing with distractions during the command. The mean of these three trials was calculated to give the mean variable value for that assessment. Percentage changes for A1-A0, A2-A0 and A2-A1 were calculated. The minimal clinically important difference (MCID) values of the kinematic variables are not yet known in the current literature.

FM-UE, ARAT, total MAS, total MRC, CAHAI and ABILHAND scores were calculated adding item values using Microsoft Excel. A1-A0, A2-A0 and A2-A1 changes were calculated. To determine clinical significance, MCID values of outcome measures were used if already described in the literature. MCID is defined as the smallest difference in score in the domain of interest that patients perceive as beneficial or that would be clinically meaningful.

All the statistical analyses were carried out in Microsoft Excel and IBM SPSS version 22 software packages. The calculations of mean, median, SD and inter-quartile ranges and drawing the chart figures were done in Microsoft Excel. Non-parametric tests for calculating data significance levels were done using SPSS. A non-parametric Friedman’s test was used to detect the significance of the three related samples A0, A1 and A2. If this test showed statistical significance, a Wilcoxon post-hoc analysis was used to test for significance between two related samples such as A0A1, A0A2 or A1A2. The significance levels for these tests were set at p = 0.05. SPSS was also used to do multiple regression analyses to test the relationships between independent variables such as baseline scores, age, time since stroke and device usage; and dependent variables such as change in outcome measures.

## Results

Nineteen participants were recruited to the study. After recruitment, two participants could not use the device in their homes and dropped out of the study. One of these two participants had reassessed his home situation (in view of some relatives living in his house for holiday) and felt there was inadequate space in his house to accommodate the device for the period of the study. The other participant was unable to move the joystick to complete computer tasks even using the full assistance mode of the device. Hence, this participant could not continue in the study. Seventeen participants completed 8-week home use of hCAAR. The demographic information of these participants at the time of starting device use is shown in Table 1.

**Table 1:** **Demographic variables of participants**

Baseline characteristics	Participants (n = 17)
Mean age in years	56.4 (11.5)
Sex	
Male	14
Female	3
Mean time since stroke in months	24.8 (17.8)
Type of stroke	
Infarction	13
Haemorrhage	4
Side of weakness	
Right dominant	9
Right non-dominant	0
Left non-dominant	8
Left dominant	0
Other deficits	
Expressive dysphasia	6
Pain in affected arm	3
Visual inattention	1
Employment	
Not in employment before stroke	13
Gave up employment since stroke	3
Employed	1

The device was installed in various locations within the participants’ homes both at ground floor and first floor levels. Ten participants had the device in their living rooms, four in their bedrooms (first floor), two in dinning rooms and one in the conservatory. The research team did not encounter any difficulties in installing the device in these locations.

After installation and retraining on the user instructions, 13 participants did not experience any difficulty in logging in and using the device independently during the entire 8-week device-use period. Two participants required help from family members during the first one week to log in and initialise the joystick, but were able to play games independently once the joystick was initialised. These two participants became fully independent in using the device after one week. Two other participants required help from family members to log in and initialise the joystick for two weeks before becoming fully independent in the using the device.

One participant could not use the device for almost the entire study period because of personal problems (total usage 12 min). Three participants were unable to use device for more than two weeks during the 8-week period due to unexpected travel and illness. One participant had a 5-year old son who would not let the participant concentrate on game play when he was around. This participant could use the device only at times when the son was asleep and consequently the usage time was affected.

No serious adverse events were observed in the study. All clinical adverse events were managed by the clinicians in the research team and did not need hospital admission or external clinician intervention during device-use period. One participant had a fall (and sustained a neck of femur fracture) after completing home-use of device. This event was deemed to be unrelated to study. This participant did need hospital admission to manage the hip fracture. Other clinical observations during the study period are listed in Table 2.

**Table 2:** **Clinical observations and adverse events**

Number of participants	Clinical observations/Clinical adverse events	Actions taken	Result
One	Wrist pain when uses joystick for more than 10 min particularly while playing higher-level games	Advised to play lower level games, reduce duration of session, use a wrist splint and do wrist stabilising and strengthening exercises.	Reduction in wrist pain
Three	Shoulder pain. Two participants reported an increase in shoulder pain with device usage. One of them was noted to be sitting with back unsupported in the chair and had excessive wrist flexion while holding the joystick. The third participant had long-standing shoulder pain unrelated to device usage.	All three participants had shoulder impingement syndrome on clinical examination. They were advised on shoulder strengthening and range of motion exercises. One participant was advised on sitting back against the chair and holding joystick handle with the wrist in a neutral position during game play.	Shoulder pain improved with exercises
One	Injured finger with bruising while trying to stretch fingers to hold the handle of the joystick	Advised on slow stretching of fingers prior to holding handle. Also received botulinum toxin injection to finger flexors as routine planned treatment unrelated to this study.	No further injury while gripping joystick
One	Reported scapula becoming more prominent in affected upper limb (has had the prominence since stroke)	Reassured and advised on scapular stabilisation exercises.	No further worsening of prominence
Four	Could not use device as expected due to personal problems or medical problems (such as chest infections) unrelated to device usage	None. Research team not made aware of personal problems by the participants during the study period.	Usage improved once medical problems were resolved
Two	Low mood. One participant due to chronic ill health and other participant due to family member being unwell.	Reassurance.	n/a
One	Painful thumb and index finger in the affected hand, reported to be not related to device usage.	Found to have osteoarthritis of small joints in these fingers. Advised to use topical analgesia.	Good relief of symptoms with topical analgesia
One	Episodes of dizziness during study period, reported to be unrelated to device use. Lacked motivation to use device.	Dizziness symptoms resolved with adjustment of his regular medications. Needed lot of encouragement from participant’s wife to use the device.	Needed encouragement from wife throughout study period

### Device-related events

All device-related events were managed by the research engineers in the research team (JG and ML) and did not need any external professional engineering input. Two joysticks needed to be changed as they became noisy and jerky in movement. Six participants encountered joystick calibration/initialising problems that led to them losing track of the joystick position on the screen while starting game play. This was resolved with a home visit by the engineer JG and additional training on initialising the joystick; the participants picked this up easily after one training session at home.

### Device usage time

The mean device usage time during the 8-week study was 520 min (range 12 min – 1468 min, SD 381 min). The median usage time was 433 minutes (IQR 250 – 791 min). One participant could not use the device beyond 12 min due to personal problems.

### Summary of kinematic and clinical outcome scores

Data were available for 17 participants who completed 8 weeks of device use. The data from two participants were not included in the analysis as there were no assessments done at one of the assessments points due to ill health on the day. The descriptive statistics for the remaining 15 participants are shown in Tables 3, 4, 5, 6 and 7. The kinematic scores at A1 in the far reach task showed statistically significant changes in movement time and path length (p < 0.05) (Table 3). The percentage improvement in the median movement time at A1 was by 19%, path length improved by 15% and jerk improved by 19% (Table 4). The improvements (except path length) were maintained at the final assessment (A2) suggesting that the improvements were retained one month after using the device. All the clinical score improvements at A1 were statistically significant when compared to baseline scores (p < 0.05) (Table 5). The average FM-UE score in this study showed a median improvement of one point at A1 (post-use assessment). The median increase in other clinical scores at A1 were 3 points in the ARAT score, 5.5 points in CAHAI, 3 points in ABILHAND and 2 points in the total MRC (Table 6). The total MAS score decreased by 1.5 points. All the improvements were maintained at the final assessment (A2) suggesting the gains were retained at one-month follow-up.

**Table 3:** **Kinematic variable scores at three assessment points (mean and standard deviation) and statistical significance values**

Kinematic variable	Baseline A0		Post-use A1		Final A2		Significance		Significance		Significance		Significance	
	Mean (SD)		Mean (SD)		Mean (SD)		A0A1A2		A0A1		A0A2		A1A2	
	Near reach	Far reach	Near reach	Far reach	Near reach	Far reach	Near reach	Far reach	Near reach	Far reach	Near reach	Far reach	Near reach	Far reach
**Movement time**	**0.48**	**0.66**	**0.42**	**0.48**	**0.37**	**0.46**	**0.105**	**0.006**	**n/a**	**0.036**	**n/a**	**0.008**	**n/a**	**0.460**
	(0.20)	(0.33)	(0.16)	(0.17)	(0.11)	(0.11)								
**Path Length**	**154.00**	**188.53**	**141.31**	**164.70**	**126.01**	**161.05**	**0.011**	**0.015**	**0.112**	**0.061**	**0.011**	**0.027**	**0.140**	**0.650**
	(51.42)	(49.5)	(39.45)	(38.35)	(18.28)	(20.75)								
**Normalised Jerk**	**393.20**	**453.83**	**276.79**	**385.62**	**282.35**	**349.68**	**0.038**	**0.091**	**0.069**	**n/a**	**0.023**	**n/a**	**1.000**	**n/a**
	(173.01)	(179.15)	(114.59)	(149.56)	(144.82)	(93.06)								

Movement time – in sec.

Path Length – in mm.

Normalised Jerk – no units.

n/a – not applicable.

**Table 4:** **Kinematic variable scores at three assessment points and percentage change in scores (median and IQR)**

Kinematic variable	Baseline A0		Post-use A1		Final A2		A1 – A0		A2 – A0		A2 – A1	
	Median		Median		Median		% change		% change		% change	
	(IQR)		(IQR)		(IQR)		Median (IQR)		Median (IQR)		Median (IQR)	
	Near reach	Far reach	Near reach	Far reach	Near reach	Far reach	Near reach	Far reach	Near reach	Far reach	Near reach	Far reach
**Movement time**	**0.43**	**0.53**	**0.41**	**0.44**	**0.33**	**0.44**	**-10**	**-19**	**-9**	**-20**	**-8**	**2**
	(0.38 – 0.51)	(0.48 – 0.63)	(0.32 – 0.49)	(0.37 – 0.55)	(0.29 – 0.43)	(0.42 – 0.55)	(-30.5 – 3.5)	(-39.5 – -11)	(-37.5 – 0.5)	(-42.5 – -8.5)	(-26 – 12.5)	(-11 – 9.5)
**Path Length**	**132.85**	**187.12**	**127.80**	**155.95**	**125.66**	**165.94**	**-4**	**-15**	**-7**	**-11**	**-** 3	**4**
	(122.18 – 172.03)	(148.89 – 212.35)	(112.83 – 154.22)	(140.40 – 180.41)	(111.56 – 135.50)	(140.23 – 179.23)	(-15 – -1)	(-19.5 – -4.5)	(-21 – -3.5)	(-23 – -4.5)	(-16 – 2.5)	(-4.5 – 5)
**Normalised Jerk**	**370.38**	**447.75**	**258.17**	**388.65**	**233.26**	**363.64**	**-23**	**-19**	**-34**	**-20**	**-7**	**-7**
	(301.71 – 405.46)	(350.22 – 488.43)	(227.38 – 283.43)	(289.28 – 468.57)	(193.63 – 308.34)	(307.53 – 391.89)	(-55.5 – 1)	(-29 – 4.5)	(-44.5 – -21.5)	(-42 – -1)	(-31 – -48.5)	(-23.5 – 13)

Movement time – in sec.

Path Length – in mm.

Normalised Jerk – no units.

**Table 5:** **Clinical outcome scores at three assessment points (mean and standard deviation) and statistical significance values**

Outcome measure	Baseline A0	Post-use A1	Final A2	Significance	Significance	Significance	Significance
	Mean (SD)	Mean (SD)	Mean (SD)	A0A1A2	A0A1	A0A2	A1A2
**FM-UE**	**28.5**	**31.1**	**31.2**	**0.028**	**0.009**	**0.094**	**0.964**
	(9.8)	(8.9)	(8.7)				
**ARAT**	**26.4**	**30.2**	**31.1**	**0.000**	**0.001**	**0.007**	**0.306**
	(19.9)	(18.9)	(20.1)				
**CAHAI**	**48.8**	**55.3**	**58.8**	**0.000**	**0.001**	**0.002**	**0.050**
	(21.7)	(20.1)	(18.8)				
**ABILHAND**	**18.2**	**22.5**	**23.8**	**0.000**	**0.004**	**0.001**	**0.154**
	(9.3)	(10.1)	(8.9)				
**Total MAS**	**11.0**	**9.1**	**8.5**	**0.000**	**0.001**	**0.004**	**0.344**
	(5.0)	(4.7)	(4.5)				
**Total MRC**	**36.2**	**39.1**	**39.6**	**0.000**	**0.011**	**0.005**	**0.202**
	(4.6)	(1.3)	(1.5)

**Table 6:** **Clinical outcome scores at three assessment points and change in scores (median and inter-quartile range)**

Outcome measure	Baseline A0	Post-use A1	Final A2	A1 – A0	A2 – A0	A2 – A1
	Median	Median	Median	Median	Median	Median
	(IQR)	(IQR)	(IQR)	(IQR)	(IQR)	(IQR)
**FM-UE**	**29**	**32**	**30**	**1**	**1**	**0**
	(19.5 – 36.5)	(28.5 – 35.5)	(28–36)	(1.0 – 4.0)	(-1.0 – 4.5)	(-1.0 – 4.5)
**ARAT**	**23**	**31**	**33**	**3**	**4**	**0**
	(9.5 – 44.5)	(16 – 46.5)	(11.5 - 49)	(1.0 – 4.0)	(1.0 – 5.5)	(-2.0 – 2.0)
**CAHAI**	**47.5**	**55**	**62**	**5.5**	**10**	**3**
	(33.3 – 65.8)	(42.5 – 71.8)	(48.5 – 68.8)	(4.3 – 8.5)	(2.3 – 13.5)	(0 – 6.75)
**ABILHAND**	**17**	**24**	**22**	**3**	**5**	**0**
	(11.5 – 24.5)	(16.5 - 31)	(18 – 31.5)	(1–5)	(1.0 – 8.5)	(-0.5 – 4.0)
**Total MAS**	**12**	**9.5**	**7.5**	**-1.5**	**-2**	**-1**
	(7.5 – 14.5)	(5.5 – 12.5)	(5.5 - 11)	(-2.5 – -0.5)	(-3.5 – -1)	(-2.0 – -1.0)
**Total MRC**	**38**	**40**	**40**	**2**		**0**
	(34.5 – 39.3)	(38.5 - 40)	(40–40)	(0 – 3.25)	(1.0 - 4)	(0 – 1.0)

**Table 7:** **Categorisation of participants based on changes in scores**

Group	Criteria	Participant ID number	(n)
**I**	MCID changes in all clinical measures FM, ARAT, CAHAI and ABILHAND	8,10 and 13	3
**II**	MCID change in at least one of the clinical measures FM, ARAT, CAHAI or ABILHAND	1, 2, 3, 5, 9, 11, 14, 18 and 19	8
**III**	MCID change in none of the clinical measures FM, ARAT, CAHAI or ABILHAND	4, 6, 7, 16 and 17	4

### Grouping of participants

The inter-quartile ranges for the kinematic and clinical scores suggest a wide distribution of values. Therefore to perform further analysis of the data, the participants were divided into three groups based on the magnitude of the observed changes (in relation to the MCID values of the clinical measures) and the uniformity of the changes across the different clinical measures (Table 7). The criteria used to categorise the participants are shown below in Table 7 (MCID values FM 7; ARAT 6; CAHAI 7 and ABILHAND 5). Kinematic variables were not considered for the categorisation, as the MCID values for kinematic measures are not yet established.

Although there was not a clinically significant improvement in the overall group in their outcome measures, three participants achieved clinically significant improvements in all four clinical outcome measures (FM-UE, ARAT, CAHAI and ABILHAND). An additional eight participants improved on at least one of the measures.

### Relationships between variables and outcomes

The multiple regression analysis, using the independent variables of age, time since stroke, device usage time and baseline scores, and dependent variable of change in scores, revealed no significant predictive relationships for age, time since stroke and device usage time. The baseline clinical scores (ABILHAND logit scores), particularly the A0 scores for far reach task, seem to be the only variable which approached significance levels for predictive relationship with change in scores (Pearson coefficient exceeding 0.50 and significance value of 0.058). The output of regression analysis is summarised in Table 8.

**Table 8:** **Regression analysis between variables and outcomes**

	Pearson correlations			Multiple regression coefficients - significance		
	A1	A1	A1	A1	A1	A1
	MT-near change	MT-far change	ABILH. change	MT-near change	MT-far change	ABILH. change
**Age**	0.30	0.01	0.36	0.13	0.62	0.62
**Time since stroke**	0.37	0.25	0.27	0.24	0.81	0.55
**Device usage**	- 0.11	- 0.03	0.46	0.47	0.34	0.98
**A0 MT-near**	- 0.43	n/a	n/a	0.19	n/a	n/a
**A0 MT-far**	n/a	0.36	n/a	n/a	0.82	n/a
**A0 ABILH.**	n/a	n/a	- 0.50	n/a	n/a	0.06

## Discussion

This research provides preliminary evidence that the hCAAR robotic device can be used safely in a home setting. Most of the previous robot studies have been conducted in research centres or hospitals and have had a therapist present with the patient in each treatment session. This is the first clinical study of its kind (excluding clinical case studies) in the literature in which the participants used a robotic device on their own in their homes with minimal supervision from healthcare professionals.

The feasibility study recruited 19 participants, of which 17 participants completed the 8 weeks of hCAAR home use. Two participants could not use the device and dropped out of the study: one of them did not have the minimal active movement required to move the joystick: and the other participant could not accommodate the device at home. This highlights that the most important prerequisites to use hCAAR are having a minimum voluntary movement in the upper limb and having a home environment suitable for device installation. The FM-UE score can be used to predict the participant’s ability to use hCAAR. The participant with an FM-UE score of 6/66 could not complete the computer tasks even with full assistance and hence had to drop out of the study. The participant with lowest FM-UE score in this group (12/66) was able to use the device. A FM-UE score of 12 could, therefore, be reasonably considered as the minimum score to be able to use hCAAR. This, however, cannot be considered as the definite minimum score for usability as there were no participants with a baseline FM-UE score between 6/66 and 12/66 in this study.

There were no serious adverse events during the hCAAR study. The musculoskeletal adverse events (shoulder pain, wrist pain) noted in this study are comparable to those seen in other robot studies [47]. General advice on the appropriate positioning of upper limb, rest, and using the available pain-free range of movements is the standard approach adopted in these studies. These musculoskeletal problems are also encountered in conventional therapy as well and similar management approaches are used.

hCAAR therapy had improved arm movement and functional ability of upper limb in this study. There was a statistically significant (p < 0.05) improvement in the mean clinical outcome scores at A1 and this improvement was retained at final assessment A2, one month after using the device. The improvements, did not reach clinical significance (observed gains at A1 were below the MCID values for the outcomes: 1 point in FM-UE; 3 points in ARAT; 5.5 points in CAHAI; and 3 points in ABILHAND). This however was not the case when individual participant results were analysed. Some participants did show clinically significant improvements in their scores. Three participants had achieved clinically significant improvement in all the four clinical scores FM-UE, ARAT, CAHAI and ABILHAND. This suggests that there may be participant characteristics that predict outcome from robotic therapy. Such a differential effect of robot therapy among the participants was also seen In the GENTLE robot study, where a group of seven out of the 20 chronic stroke participants showed clinically significant improvement across all outcome measures [48]. Further work needs to determine the characteristics that predict a favourable outcome.

The changes in outcome scores seen in the hCAAR study are comparable to those seen in previous robot studies. The mean improvement in FM-UE score with hCAAR was 2.5 points; this change is similar to changes ranging from 2.8 to 5.3 that have been reported in previous robot studies [49–54]. The median improvement in ARAT score with hCAAR was 3 points; this is less when compared to a 9 point change in median improvement reported in the HapticMaster robot study [55]. The mean ABILHAND logit score improved by 0.56 with hCAAR that is higher when compared to the observed change of 0.25 logits in the Bi-Manu-Track device study [54]. There was a 19% reduction in mean movement time with hCAAR whereas a 35% reduction in mean movement time was observed in a group of participants with chronic stroke in the BFIAMT robot study [53]. There were modest changes in strength and spasticity with hCAAR similar to changes reported in studies of other robotic rehabilitation devices [52, 53, 56].

The hCAAR study showed statistically significant improvement in two functional activity-based outcome measures CAHAI and ABILHAND in some participants. This is contrary to findings in the systematic review of robot studies that did not find evidence of changes in functional activities (based on changes in the FIM score) [15]. Two reasons could be identified for this finding; first, CAHAI and ABILHAND are more responsive measures than the FIM motor in upper limb motor recovery; and second, previous robot studies generally report results for the entire study cohort and do not often report on outcome measures of each individual participant.

hCAAR seemed to be most suitable for individuals with moderate arm weakness. This median baseline FM-UE score of participants was 29 (range 12–43). The individuals with severe weakness might not be best suited to use the device as suggested by the one drop-out from the study (with an FM-UE of 6/66). Participants with mild weakness might not find the device useful as they need to practice complex three-dimensional functional movements, which hCAAR is unable to provide. This finding is supported by some other robot trials where individuals with moderate impairments (score of 15 – 40 on FM-UE score) benefitted more than those with severe weakness of the upper limb [48, 50, 57–61].

In the hCAAR study, among the participants with moderate weakness, the ones with lower baselines scores seem to have better gains from device use. The regression analysis that showed that A0 baseline ABILHAND logit score was the only variable to approach predictive significant relationship (p = 0.06) with change in score value (A1-A0). A similar finding was observed in the ARMin robotic study where gains were particularly increased in participants with severe impairment at baseline [47].

The usage time in the hCAAR study was considerably lower than that of most previous robot studies. The mean usage of hCAAR was 520 min (range 12 min – 1468 min) during the 8-week period. This is lower than the usage time reported in other studies, which involved usage time of 900 to 2160 min spread over 4 – 12 weeks [47, 53, 62–64]. It can be argued that hCAAR usage time might have been sub-therapeutic and that could explain why no dose–response relationship was seen. Previous robot studies have also identified that there is no advantage of robotic therapy at a low utilisation [15, 48, 65, 66]. One trial comprising 9 hours [540 min] of conventional functional retraining did not show any benefit in chronic stroke subjects with moderate upper limb impairment [66]. This conventional retraining study however does not report whether a subgroup of participants showed improvement in upper limb function.

There was a lack of a significant predictive relationship between time since stroke and the improvement in outcomes. The mean time since stroke for the three participants who showed clinically significant improved in all four outcome measures was 11.8 months. It is an encouraging finding that hCAAR therapy can lead to clinically significant improvements in individuals in the chronic stages after stroke. It was difficult to compare the effect of hCAAR between subacute and chronic stages after stroke as most of the participants in the study were in the chronic stage. The hCAAR study failed to show any predictive value of age, time since stroke, or device usage (time) in determining the treatment effect. A similar finding has also been observed in a larger study involving 38 chronic stroke participants who used the ARMin robot for 8 weeks [47]. The authors of the ARMin trial performed a post-hoc analysis stratified by age, hand dominance and time since stroke and did not find any significant relationships between these variables and the gains.

The improvements seen at A1 in this study are sustained at the 1-month follow-up at A2. Previous robot studies suggest that improvement in chronic subjects is maintained for up to 3 months [22, 56, 67]. Robotic therapy in chronic stroke shows faster gains when compared to intensive conventional physiotherapy, but only while using the device and the gains become similar to intensive conventional physiotherapy in the long term (6 months) [47, 64]. It is encouraging to find that the short- and long-term effects of robot therapy are at least similar (if not superior) to intensive conventional physiotherapy. The long-term retention effects of hCAAR therapy need to be further researched.

Most robot studies so far have involved high-cost complex devices with therapists being involved in delivering each session of robot therapy. The only large-scale economic analysis study involving the MIT-Manus robotic device concluded that there was no increased cost-effectiveness with robot therapy when compared to intensive conventional therapy [68]. The cost of the robotic device was US$ 230,750 with additional maintenance costs (US$15,000) and cost of therapist time (US$120 for 15 min of therapist contact time per session) [68]. The cost of the hCAAR device is much lower (approx 5,000 GBP or US$ 8,400) compared to this and there is no therapist time involved for each session. A cost-effectiveness analysis in comparison to conventional therapy needs to be done in future hCAAR studies in the home setting.

### Limitations

There are some limitations to hCAAR device and the feasibility study. Firstly, hCAAR is a planar robot providing exercises only to the proximal muscles of the upper limb. The current literature suggests that the benefits to proximal muscles from exercises do not extend to the distal muscles in the chronic stage of recovery. This finding has led to the development of additional distal modules for many robotic devices, such as MIT-Manus, GENTLE and ARMin [47, 69–72]. The ADLER, RUPERT and ARMin devices are robotic devices that promote the upper limb to do real world tasks [18, 47, 73]. However these devices are too complex to be used in home settings and use of the device needs assistance from a helper or therapist. hCAAR was designed with home use in mind and there was a need to keep the device as simple as possible. The provision of additional attachments/modules would make the device bigger and more complex making it less appealing for home use.

An element of bilateral therapy has been incorporated in hCAAR; its actual contribution to motor recovery in this study is, however, minimal. Evidence on the amount and type of involvement of the unaffected limb in bilateral therapy is lacking. Most robotic devices promoting bilateral therapy such as MIME, BATRAC, BFIAMT, provide symmetrical bilateral therapy and one robot study did not show any benefit of bilateral therapy over unilateral therapy [57]. Moreover, the criticism of this approach has been that unlike the above robots, most daily activities are asymmetrical in nature. Bilateral asymmetrical therapy using robot devices needs to explored in future studies. Even though the bilateral therapy of hCAAR is asymmetrical and minimal, it is difficult to establish whether the activity of the unaffected upper limb (operating a switch) had any role in the gains observed in the study.

There was no mandatory minimum recommended usage time planned for this study. Even though participants were advised to use the device for at least 30 min every day for five days a week, the device software did not provide feedback on usage time to the participants during the study period. Lack of such reminders could have influenced device usage time in the study. Several participants suggested that the games lacked complexity and did not match their preferences. This could be one of the reasons for the low device usage time when compared to other robot studies.

The small sample of participants limits the generalisation of the results on efficacy. The aim of the feasibility study was primarily to test whether the robot device could be used safely in a minimally supervised home setting. The efficacy data shows the potential for therapeutic effect in some participants and this needs to be explored in a future hCAAR study in a larger sample of participants.

Participants in the hCAAR study, even though they had a wide range of impairments, did not include individuals with significant visual field defects, severe language impairments, or those with severe mobility limitations. The selection of participants was influenced by the nature of the study in which the participants needed to be able to attend the research laboratory (using their own transport) for the introduction to the device and the outcome score assessments. Future hCAAR studies must include individuals with greater disability to test usability by them. Suitable outcome measures need to be chosen so that they can be completed at homes and avoid participants having to visit the research laboratory for the assessments.

This study had a greater number of male than female participants (14:3) and greater number of middle aged than elderly participants. Only three participants were above 65 years of age and only one participant was above 70 years of age. This limits the assumptions we could make on whether hCAAR would be equally usable by females and elderly people. However, this study included one female participant who was 81 years of age and who had never used a computer in her life before. She needed some supervision from family members to use hCAAR at first but became independent thereafter and completed the study with reasonable usage time in the 8-week period (461 min). This example suggested that the device has the potential for use by elderly patients.

This study lacked multiple baseline assessments to estimate ongoing natural recovery. From the spontaneous recovery studies reported by Duncan et al. we know that the recovery pattern tends to plateau after 3 or 6 months, depending on the severity of the stroke [74]. In this study, most of the patients were in the chronic phase of recovery (mean time since stroke 24.8 months; median 26 months) and there was a definite improvement in outcomes scores at A1 followed by a plateau or slight dip in improvement at A2. This improvement pattern suggests that the observed changes are due to hCAAR use in the intervention period and also suggests that with the aid of rehabilitation treatments, motor improvements can occur beyond the 6 months post-stroke period.

## Conclusions

In conclusion, the hCAAR feasibility study was the first clinical study of its kind reported in the literature; in this study, 17 participants used the robotic device independently for eight weeks in their own homes with minimal supervision from healthcare professionals. Statistically significant improvements were observed in the kinematic and clinical outcomes in the study.

In the future, the hCAAR games could be improved and the feedback the device provides to the user on their results and performance needs to be developed. Internet linkage to a remote therapist to monitor the therapy and provide professional feedback must also be considered. A future clinical study would need to explore the use of hCAAR in a larger, more heterogeneous sample of participants in the home setting. A study design comparing the combination of conventional therapy and hCAAR with conventional therapy alone needs to be explored. A combination of outcome measures that span the domains of the ICF framework needs to be included in any future study.

## Authors’ original submitted files for images

Below are the links to the authors’ original submitted files for images. Authors’ original file for figure 1 Authors’ original file for figure 2 Authors’ original file for figure 3
