# Supplementary figures and images for: Home-based Computer Assisted Arm Rehabilitation (hCAAR) robotic device for upper limb exercise after stroke: results of a feasibility study in home setting
Source: J Neuroeng Rehabil. 2014 Dec 12;11:163. doi: 10.1186/1743-0003-11-163 (PMC4280043; doi:10.1186/1743-0003-11-163)

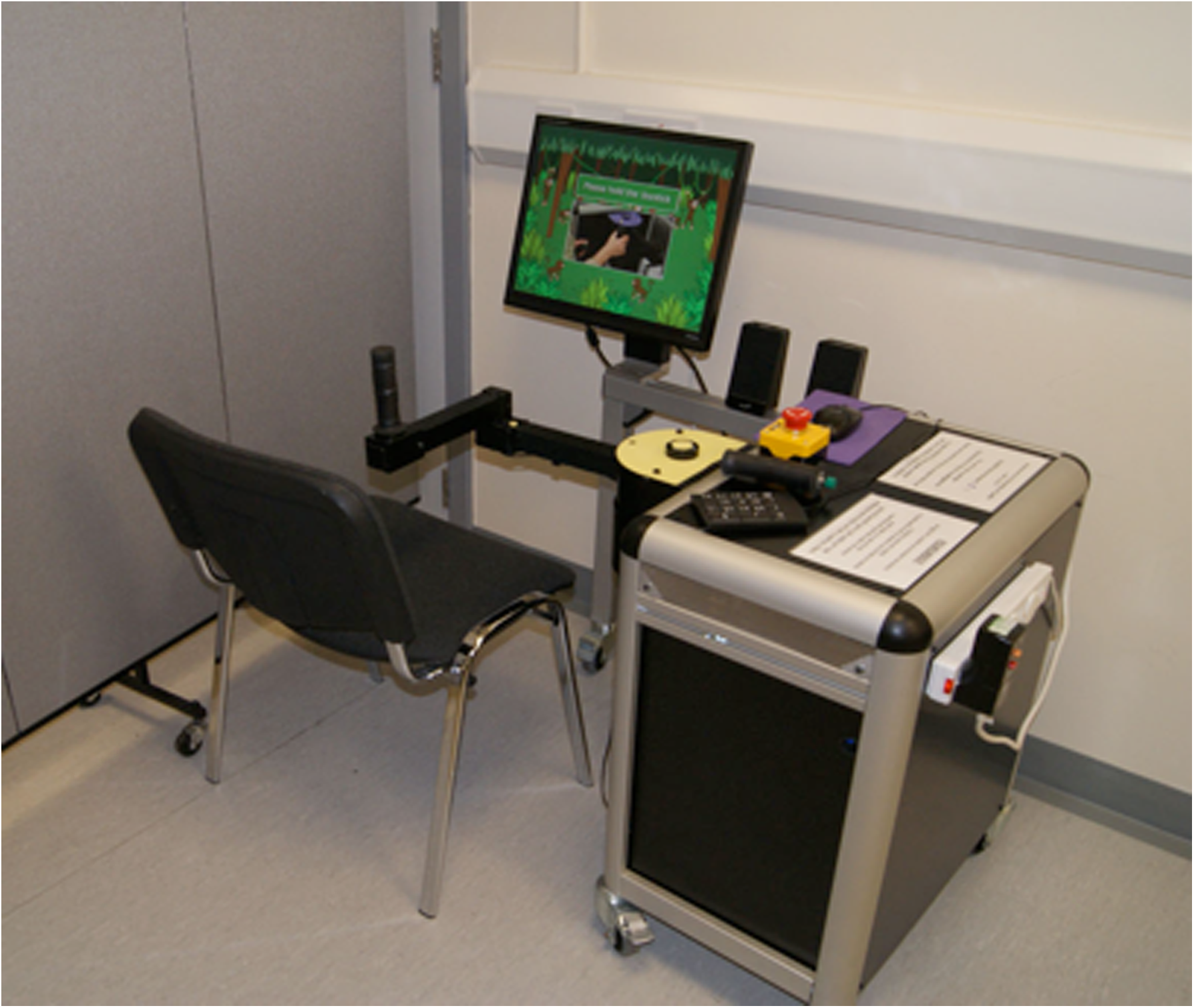

Supplement: Supplementary file 1 — Authors’ original file for figure 1 [file 12984_2014_686_MOESM1_ESM.tif]

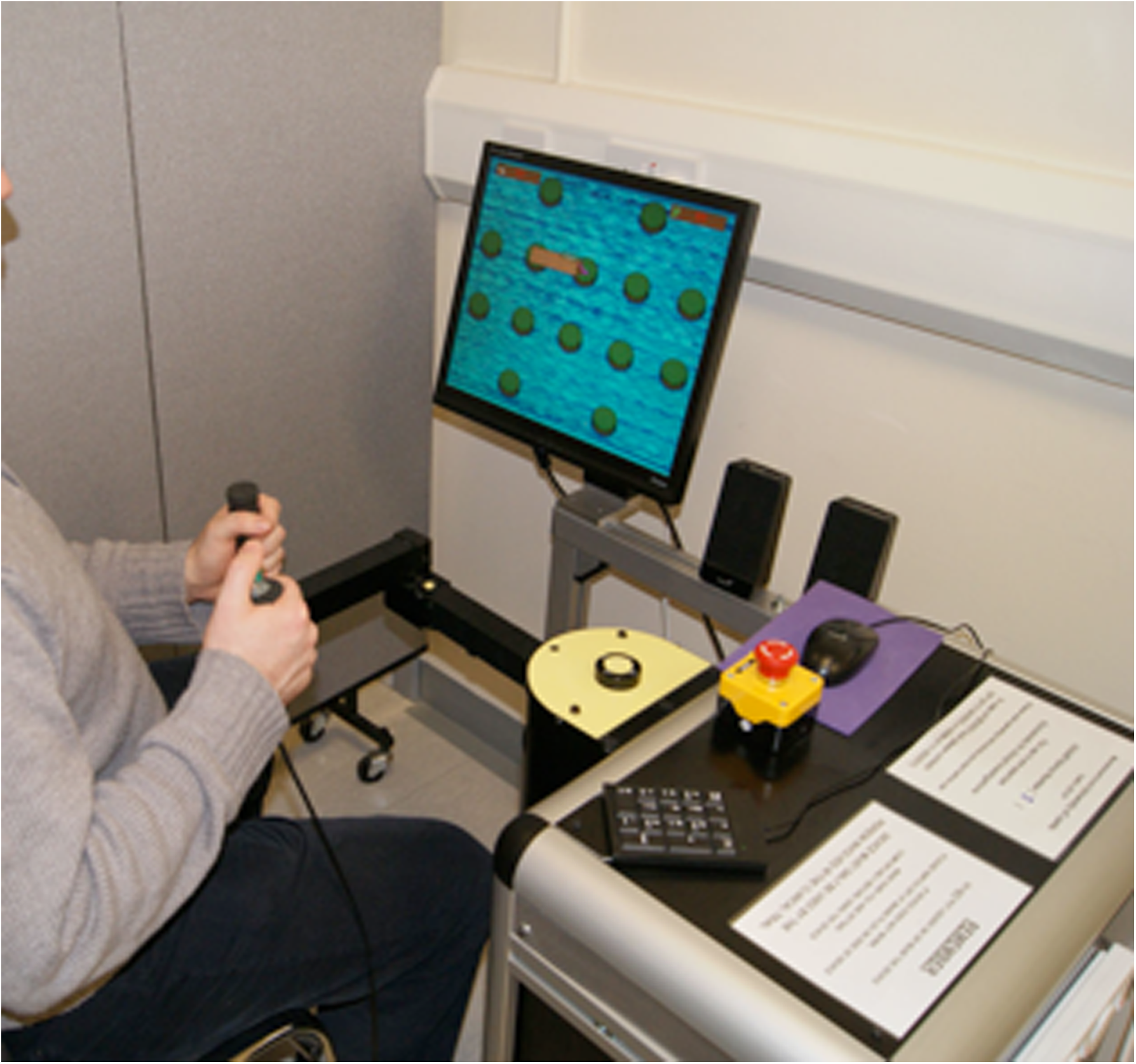

Supplement: Supplementary file 2 — Authors’ original file for figure 2 [file 12984_2014_686_MOESM2_ESM.tif]

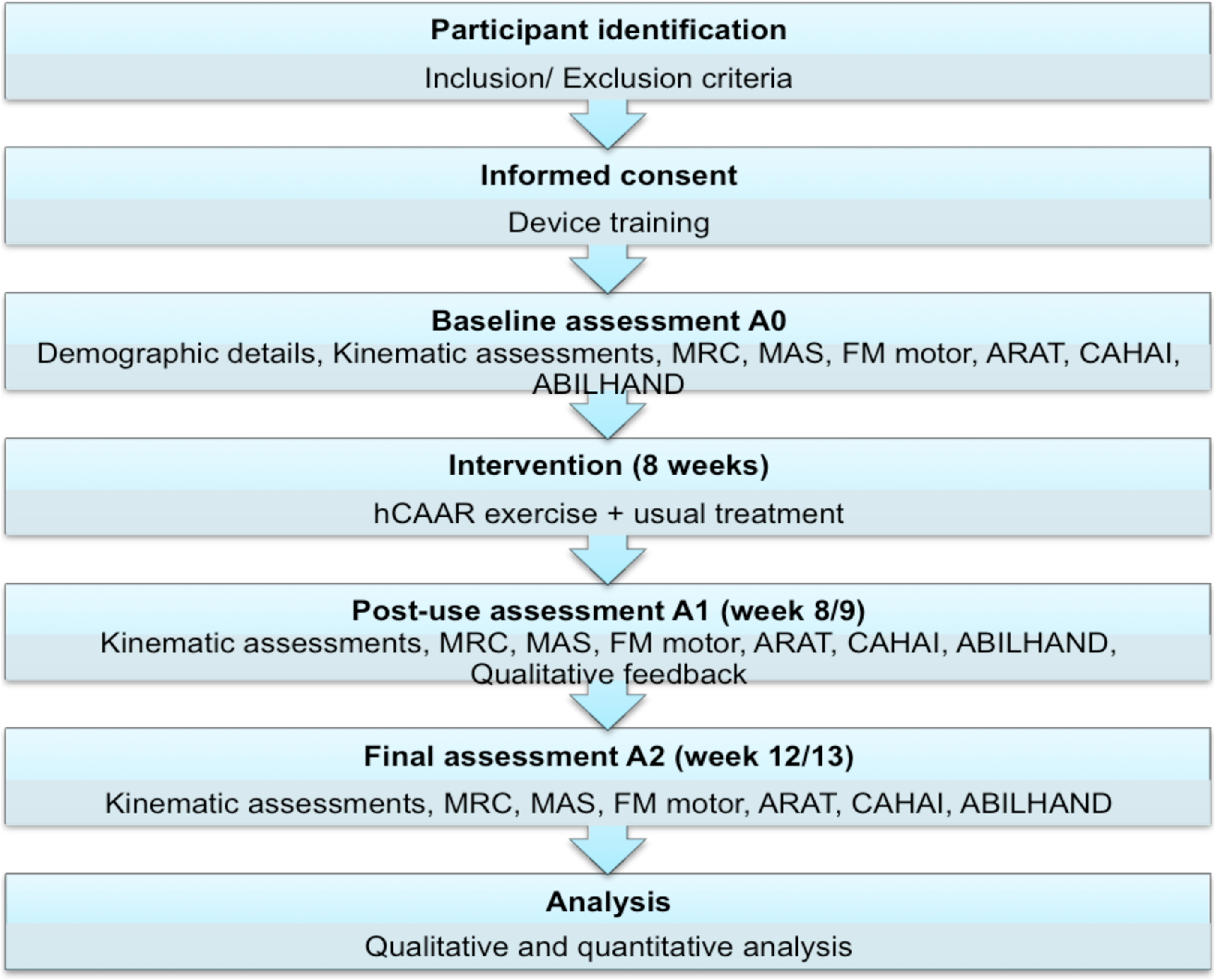

Supplement: Supplementary file 3 — Authors’ original file for figure 3 [file 12984_2014_686_MOESM3_ESM.tif]
